# Regulation of T Cell Activation and Differentiation by Extracellular Vesicles and Their Pathogenic Role in Systemic Lupus Erythematosus and Multiple Sclerosis

**DOI:** 10.3390/molecules22020225

**Published:** 2017-02-02

**Authors:** Cristina Ulivieri, Cosima T. Baldari

**Affiliations:** Department of Life Sciences, University of Siena, Via Aldo Moro 2, 53100 Siena, Italy; cosima.baldari@unisi.it

**Keywords:** autoimmunity, systemic lupus erythematosus, multiple sclerosis, immunological synapse, Th17 cells, regulatory T cells, extracellular vesicles

## Abstract

How autoreactive tissue-infiltrated effector T cells are induced and sustained in autoimmune disease, usually dominated by the Th1 and Th17 subsets, is still largely unknown. In organ-specific autoimmunity, self-reactive T cells initially activated by dendritic cells (DCs) in the lymph nodes migrate and infiltrate into the target tissues where their reactivation by peripheral tissue antigen is a prerequisite for effector cytokine production and tissue destruction. The target tissue microenvironment, as well as the local microenvironment at the immune synapse formed by T cells that encounter cognate antigen presenting cells (APCs) shave recently emerged as critical factors in shaping the differentiation and function of self-reactive effector T cells, providing the signals required for their activation in the form of the self-antigen and cytokine milieu. Moreover, depending on the specific microenvironment, self-reactive effector T cells have the ability to change their phenotype, especially Th17 and regulatory T (Treg) cells, which are characterized by the highest instability. In this context, cell-derived extracellular vesicles, i.e., vesicles carrying cytosolic proteins and nucleic acids protected by a phospholipid bilayer, as well as membrane-associated proteins, with the ability to spread throughout the body by means of biological fluids, are emerging as key mediators in intercellular communications and in the modulation of the microenvironment. In this review, we will discuss recent findings implicating extracellular vesicles (EVs) at different steps of CD4^+^ T cell differentiation to specific effectors, with a focus on the Th17/Treg balance and its alterations in systemic lupus erythematosus and multiple sclerosis.

## 1. Introduction

Autoimmune diseases are a heterogeneous group of disorders subdivided into systemic and tissue-specific diseases. While the initiating event is still largely unknown, the breakdown of self-tolerance and aberrant induction of the immune response to self-antigens are the acknowledged disease-causing features of all autoimmune disorders. Research efforts dedicated to dissecting the pathogenic mechanisms of autoimmunity have led to the identification of predisposing genetic risk factors shared by several autoimmune diseases; however, the results obtained from genome-wide association studies have clearly demonstrated that both environmental and epigenetic factors contribute to the initiation and progression of individual autoimmune diseases [1]. In this context, it becomes clear that the environment, including infections, diet, climate, socioeconomic status and stress, can contribute to shaping the immune system by acting on genetic susceptibility profiles [2].

At the cellular level, autoimmune diseases are characterized by an enhanced frequency of autoreactive Th1/Th17 effectors cells paralleled by a decrease in the frequency of Treg cells. Accumulating evidence suggests that the resistance of Th1/Th17 cells to regulatory T (Treg) cells suppression might also contribute to the pathogenesis of autoimmunity [3]. While intracellular signaling molecules downstream of the T cell receptor (TCR) contribute to the alterations in the Th1/Th17/Treg balance, the microenvironment plays a central role, as witnessed by the impact of the specific cytokine milieu, provided by innate immune cells and effector T cells, on T cell differentiation and function/stability, both at the onset and during autoimmune disease progression [4]. Recently, extracellular vesicles (EVs) have emerged as key mediators in intercellular communications. These vesicles, released by a variety of cells into the extracellular environment, are lipid bilayer structures containing cytosolic molecules of the parental cells, including proteins, mRNAs, long non-coding RNAs and microRNAs (miRNAs). As such EVs, have the potential to profoundly modulating the microenvironment [5]. Characterizing the contribution of EVs to the autoimmune microenvironment may help to identify new therapeutic targets.

## 2. The Microenvironment of the Immune Synapse Cleft Controls Helper T Cell Differentiation

Naive CD4^+^ T cells have the potential to differentiate into multiple effector T helper (Th) cells depending on TCR signal strength and on the cytokine milieu, which is mainly shaped by innate immune cells [6]. In this context, dendritic cells (DCs) represent master regulators of effector T cell responses to invading pathogens. DCs can indeed instruct T cell polarization by providing proper antigen-dependent TCR stimulation via major histocompatibility complex (MHC) molecules, as well as costimulation through surface receptors, which are upregulated on the DCs surface following pattern recognition receptor engagement by pathogen-associated molecular patterns. In addition, according to the qualitative/cytokine model of differentiation [7], DCs have the potential to instruct T cell differentiation by altering the microenvironment through the release of specific cytokines, including interleukin (IL)-12, IL-4 or IL-6 and Transforming Growth Factor β (TGF-β), which are Th1-, Th2- and Th17-polarizing cytokines, respectively.

The role played by the cytokine milieu in T cell polarization has been recently revisited based on evidence that the strength of TCR signaling, which depends on the quality and the quantity of antigen presented by DCs during T cell-DC interaction, as well as costimulatory signals, control the expression and polarization of cytokine receptors towards the immunological synapse (IS) [8]. Hence, the ability of T cells to respond to cytokines released by DCs relies on early signaling at the IS, which suggests that orchestration of Th cell differentiation by cytokines represents only the second step of this differentiation program. Based on in vitro and in vivo studies, it has been demonstrated that Th1 cell development is favored by strong TCR signals and the formation of a long-lasting mature IS, which in turn promotes IL-12 receptor subunit beta-2 (IL-12Rβ2) and interferon (IFN) γ receptor (IFNγR) polarization to the T cell-DC contact site [8,9,10,11]. At variance, Th2 polarization is favored by weak TCR signals and brief T cell-DC interactions [8]. Of note, while the polarized secretion of IL-12 by DCs is required for efficient IL-12-dependent signaling leading to Th1 cell differentiation [9], IL-4 receptor (IL-4R) polarization towards the IS is not required for Th2 cell differentiation [11]. Intermediate-to-strong TCR signals in the presence of IL-6 and TGF-β have also been associated with Th17 cell differentiation; however, the contribution of IS stability, as well as the role of directional secretion of IL-6 remain to be elucidated [7,12]. In this respect, it should be pointed out that low-strength TCR signals and CD28 costimulation had been previously reported to promote Th17 cell differentiation [13]. In agreement with the key role of TCR signal intensity in addition to the cytokine milieu, it has been shown that cholera toxin (CT), an exotoxin produced by *Vibrio cholerae*, promotes Th17 differentiation, mainly by lowering the strength of TCR signaling, which results in lower IL-2 production, besides promoting DC secretion of Th17-polarizing cytokines [14].

**Figure 1:**
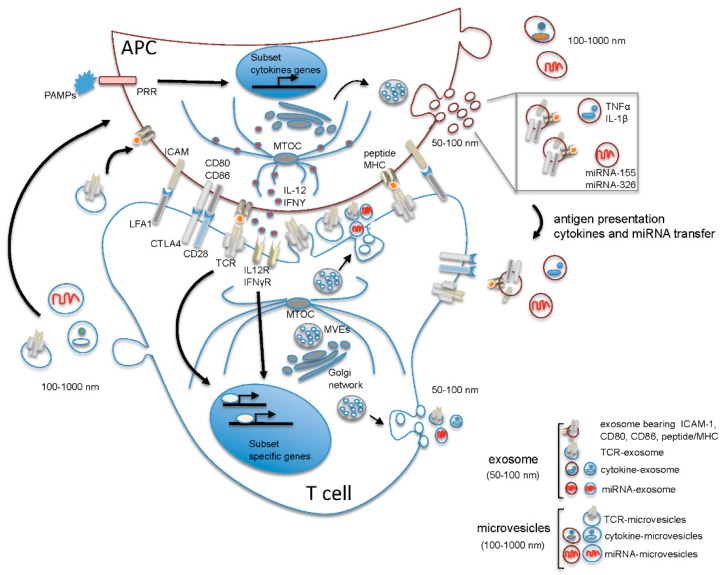
Suggested model of polarized and non-polarized extracellular vesicle (EV) release during T cell-antigen presenting cells (APC) interaction. Upon contact of the T cell receptor (TCR) with peptide/major histocompatibility complex (MHC) presented by the APC, the segregation of molecules that participate in cell activation occurs at the T cell-APC contact, resulting in the formation of the immune synapse, a highly organized structure characterized by the central accumulation of TCR and peptide/MHC on the T cell and APC side, respectively, and by the formation of a peripheral ring of adhesion molecules (the major being leukocyte function-associated antigen 1 (LFA-1) on T cells and intercellular adhesion molecule 1 (ICAM-1) on APCs), which contribute to consolidating the interaction between T cell and APC leading to the formation of a mature synapse. Intracellularly, the polarization of the microtubule-organizing center (MTOC) to the contact site drives polarized membrane trafficking towards the immunological synapse (IS) and contributes to spatially organize the intracellular signaling and the polarized secretion of soluble mediators into the synaptic cleft. MicroRNA (miRNA)-exosomes and TCR-microvesicles are released from Th cells into the synaptic cleft in a polarized manner, while APC-derived microvesicles and exosomes are released outside the synaptic cleft. Of note, in Th cells, multivesicular endosomes (MVEs) from which exosomes originate are positioned near the contact zone, while in APCs, MVEs do not polarize towards the contact zone, and the release of exosomes and microvesicles occurs in a non-polarized manner. The release of exosomes and microvesicles from T cells outside the synaptic cleft is also shown. Note that the content of EVs has been simplified showing in each vesicle only one of the known components. APC: antigen presenting cell; CD28, CD80, CD86: cluster of differentiation (CD) 28, 80, 86; CTLA4: cytotoxic T-lymphocyte antigen 4; ICAM: intercellular adhesion molecule 1; IL-1β: interleukin 1 beta; IL-12: interleukin 12; IL12R: interleukin 12 receptor; IFNγ: interferon gamma; IFNγR: interferon γ receptor; LFA-1: leukocyte function-associated antigen 1; miRNA: microRNA; MTOC: microtubule-organizing center; MVE: multivesicular endosomes; PAMPs: pathogen-associated molecular pattern molecules; peptide MHC: peptide loaded major histocompatibility complex; PRR: pattern recognition receptor; TCR: T cell receptor; TNFα: tumor necrosis factor alpha.

Collectively, these data support a quantitative model where naive CD4^+^ T cell commitment is first dictated by the duration of the interaction between T cell and DC, which is initially dependent on the avidity and quantity of antigen presented by DC and taken over by cytokines only subsequently, when TCR-dependent polarization of cytokine receptors to the IS has occurred. Cytokine-mediated control of effector T cell differentiation is also regulated by the polarized secretion of cytokines at the IS by the DC, at least in the Th1 differentiation program, indicating that both T cells and DCs contribute to generating a specific IS microenvironment that drives Th cell differentiation [15,16] (Figure 1).

The discovery that T cells are able to release extracellular vesicles into the synaptic cleft has added a further level of complexity to the molecular events governing Th cell differentiation. EVs are lipid-bilayer vesicles of sizes ranging from 50–1000 nm released into the extracellular milieu by almost all nucleated cells. Among immune cells, EVs were first discovered in B cells and DCs in the 1990s, but have only recently attracted much attention due to their emerging role in intercellular communication [5]. Based on their origin and size, EVs have been classified into microvesicles (100–1000 nm) originating by blebbing of the plasma membrane of donor cells and exosomes (50–100 nm), which are formed in multivesicular endosomes (MVEs) and are released from donor cells by exocytosis. In addition, larger vesicles (1–5 µm) released as blebs of apoptotic cells have been described and named apoptotic bodies [17]. These small phospholipid-membrane vesicles carry both surface molecules and cytosolic contents of the donor cells, including protein and RNA, and have the potential to modulate the response of the target cells depending on their composition. While the molecular mechanisms governing their biogenesis, as well as their specific composition has begun to be addressed, the precise mechanism of EV entry into the target cells requires further investigation [18]. 

The importance of EVs in shaping the microenvironment is only beginning to emerge, including in the context of the IS. The release of TCR-enriched exosomes from T cells following stimulation with anti-CD3 monoclonal antibodies (mAbs) was previously reported [19]. Using supported lipid bilayers containing intercellular adhesion molecule 1 (ICAM-1) and peptide/MHC, Choudhuri et al. recently [20] found that TCR-enriched vesicles budding from T cells are released into the IS cleft. The ability of T cells to release TCR-containing vesicles was further confirmed in conjugates with antigen-loaded B cells. Moreover, using bilayers containing TCR-enriched microvesicles and B cells presenting cognate peptide/MHC complexes, they found that these TCRs are transferred to the B cells where they remain activation-competent, suggesting a novel form of contact-independent antigen presenting cell (APC)-T cell crosstalk. Antigen-dependent transfer of exosomes containing miRNA from T cells to APC during IS assembly has also been documented [21], indicating that EVs might represent a novel and efficient way for the transfer of genetic information (Figure 1). Interestingly, both the functionality of the miRNA and the efficiency of the transfer appear to be dependent on the formation of a mature IS [21], suggesting that the intimate contact between APC and T cells is required not only to spatially reorganize surface molecules in order to set up a productive IS, but also to create a permissive microenvironment instrumental for T cell activation. It should be underlined that the finding that polarized EV release occurs at the IS does not rule out that multidirectional release of T cell-derived exosomes and microvesicles might additionally take place.

In agreement with the view that T cells instruct APC via EVs during IS formation, EV release from the APC into the synaptic cleft has not been documented. Furthermore, it has been demonstrated that MVEs from which exosomes are generated polarize together with the microtubule-organizing center (MTOC) towards the IS on the T cell side, but do not translocate to the apposed membrane in APCs despite the fact that the MTOC polarizes towards the IS also in these cells [9,22]. It should however be underscored that APC-derived EVs have the potential to modulate T cell function and differentiation by exposing on their surface MHC-peptide complexes, as well as costimulatory molecules, such as CD80, CD86 and ICAM-1, which are essential for the activation of CD4^+^ T cells by cognate peptide-loaded MHC complex (pMHC) [23]. These APC-derived exosomes promote T cell activation by favoring the exchange of MHC-peptide complexes between APCs both in vitro and in vivo and as such increase the availability of antigen-loaded APC [24,25,26]. This suggests that, while APC-derived exosomes do exert a T-cell modifying function, this occurs mainly outside the synaptic cleft (Figure 1). Hence, both the polarized release of T cell-derived microvesicles and exosomes and the multidirectional release of APC-derived EVs contribute to modifying the IS microenvironment. Based on these findings, the synaptic cleft shaped by both T cells and DCs emerges as a highly specialized microenvironment instrumental to naive CD4^+^ cell fate determination (Figure 1).

## 3. Role of EVs in Th17/Treg Cell Differentiation and Function

An altered balance between Th17 and Treg cells has been associated with several autoimmune diseases, with Th17 cells playing a central role in their pathogenesis by means of their ability to release large amounts of the pro-inflammatory cytokine IL-17 [4], and Treg cells acting as the gatekeeper of tolerance, which is defective in autoimmunity. Treg cells can indeed directly suppress T cells, thereby preventing unwanted/exaggerated immune responses, by a plethora of mechanisms, including the release of immunomodulatory cytokines (IL-10, TGF-β, IL-35), the expression of the interleukin-2 receptor alpha chain (CD25), which effectively competes for IL-2 binding by effector T cells, and the expression of galectin-1, which induces cell cycle arrest. Moreover, Tregs can suppress effector T cells indirectly by impairing the function of APC through the expression of the ectonucleotidases CD39 and CD73, which catalyze the production of the immunosuppressive molecule adenosine, by cytotoxic T-lymphocyte antigen 4 (CTLA4)-mediated downregulation of the co-stimulatory molecules CD80 and CD86 and by preventing DC maturation through lymphocyte activation gene 3 (LAG-3), which binds MHC class II molecules expressed by immature DCs [27]. In addition, Treg cells have been demonstrated to release higher numbers of EVs compared with the other CD4^+^ T subsets following TCR engagement [28]. Interestingly, Okoye et al. [28] found that Treg-derived CD63-positive EVs were enriched in miRNAs with both anti-proliferative and pro-apoptotic properties, miR-466, miR-195 and miR-16 being the most abundant among the miRNAs expressed by parental Treg cells. Furthermore, using Dicer^−/−^ conventional T cells in coculture with Treg cells, they demonstrated miR-155, miRNA Let-7d and Let-7b transfer from Treg to conventional T effector cells. Transfection of Th1 cells with Let-7d, but not with miRNA-155 or Let-7b, results in reduced Tumor Necrosis Factor (TNF) and IFN-γ mRNA levels, Th1 cell proliferation and IFN-γ secretion, suggesting that EV-mediated transfer of the miRNA Let-7d from Treg to conventional T cells might be an additional mechanism of T cell suppression by Treg cells. The importance of Let-7d in Treg EV-mediated suppression of Th1 cell proliferation was further demonstrated in vivo by transferring Treg cells, which released Let-7d-depleted EVs in a mouse model of colitis. The presence of the CD73 ectoenzyme, but not of CTLA4, on EVs released by Tregs following TCR engagement has been moreover associated with the suppressive activity of this population [29], indicating that EVs released by Treg cells might contribute to the suppression of effector T cells by affecting the local microenvironment, as well as by directly modulating the expression of specific mRNAs in the target cells through their miRNA cargo. Of note, the finding that the Treg cells have a higher suppressive activity compared with Treg-derived EVs suggests that both the release of EVs and additional contact-dependent mechanisms are required for the efficient control of T cell responses [28].

The presence of immunomodulatory cytokines in Treg EVs has not been documented to date; however, TGF-β has been found in EVs derived from DCs modified to express TGF-β1, and it has been demonstrated that, unlike soluble TGF-β1, TGF-β1-EVs play a protective role during inflammatory bowel disease development by promoting CD4^+^Foxp3^+^ Treg cell development and decreasing the proportion of Th17 cells [30]. Interestingly, the stability of TGF-β1 stored in EVs is higher compared with the soluble form, further indicating that these vesicles might prevent degradation of cytokines, thereby favoring their function far away from the parental cell. Recently, the same group [31] found that EVs with high levels of TGF-β1 were released from intestinal epithelial cells under physiological conditions and contribute to maintain intestinal tract immunotolerance, since the transfer of these EVs into inflammatory bowel disease mice decreases disease severity by promoting development of Tregs and immunosuppressive DCs. In agreement with the direct effect of TGF-β1 EVs derived from DCs on Treg cell differentiation and function, Yu et al. [32] demonstrated that treatment of experimental autoimmune encephalomyelitis (EAE) mice with EVs carrying membrane-associated TGF-β1 corrects the imbalance between Th17 and Treg cells by preventing Th17 cell development and promoting Treg cell expansion. This effect was not observed when mice were treated with EVs carrying soluble TGF-β1. Although a difference in the efficiency of EV isolation from the two parental cells cannot be excluded, these results suggest that TGF-β1 association with the EV membrane is required for their immunomodulatory function.

The key role played by TGF-β1-EVs on the development and function of Tregs has been demonstrated also in cancer, where the enhanced frequency and suppressor function of Treg cells is responsible for tumor immunotolerance. Sera of cancer patients, but not of healthy controls, are indeed highly enriched in EVs carrying immunomodulatory molecules, including TGF-β1 [33]. In this context, colorectal cancer cell- and nasopharyngeal carcinoma-derived EVs have been found to contain TGF-β1, which is in turn responsible for the expansion and suppressive function of Treg cells in vitro and in vivo [34,35,36]. Moreover, breast cancer cell-derived EVs have been shown to suppress T cell proliferation through TGF-β [37]. Interestingly, Wieckowski et al. [38] found that EVs derived from the head and neck squamous cell carcinoma cell line, PCI-13, but not EVs released by DC from healthy donors, induced Treg expansion in vitro, further underscoring the ability of tumor cells to escape tumor-specific immune responses by controlling the tumor microenvironment through EV secretion, thereby impairing the function of T effector cells. 

In addition to cytokines, miRNAs have been implicated in the control of Th17/Treg cell differentiation and in the pathogenesis of several autoimmune diseases [39,40]. In this context, multiple lines of evidence indicate that extracellular miRNAs, which circulate in body fluids by means of EVs play a key role in shaping the microenvironment [41]. Recently exosomal miR-24-3p, miR-891a, miR-106a-5p, miR-20a-5p and miR-1908 released by the nasopharyngeal carcinoma line TW03 were found to be enriched in the serum of nasopharyngeal carcinoma patients and to inhibit Th1 and Th17 cell differentiation while promoting Treg cell differentiation by decreasing the activity of extracellular signal-regulated kinases (ERK), signal transducer and activator of transcription (STAT) 1 and STAT3 and increasing the activity of STAT5 in exosome-treated T cells [42,43].

miRNA-155 has been reported to promote the development of Th1 and Th17 cells in autoimmune inflammatory diseases, including multiple sclerosis (MS) and the corresponding EAE mouse model, and silencing of miRNA-155 in mice has been shown to ameliorate EAE [44,45,46]. The negative regulators of cytokine signaling SOCS1 and SHIP1 have been identified as miRNA-155 targets [47,48], and plasma miRNA-155 was found to be associated with EVs [49]. Both miRNA-326 and miRNA-301a have been associated with MS pathogenesis due to their ability to drive Th17 cell differentiation through targeting ETS1 transcription factor, a negative regulator of this process, and the protein inhibitor of activated STAT3 (PIAS3), an inhibitor of the STAT3 pathway, respectively [50,51]. Of note, miRNA-326 has been recently also found in EVs derived from an esophageal cancer cell line [52]. 

Overall, these findings suggest that EV-mediated delivery of specific miRNAs and proteins to both naive CD4^+^ T cells and Th cells, as well as to other immune cells, including DCs, which are responsible for the production of polarizing cytokines, might impact Th differentiation and plasticity. It should however be pointed out that at present, data on the role of EV-associated miRNA/proteins circulating in the body fluids in autoimmune disease and cancer are mostly associative. 

## 4. Contribution of EVs to Autoimmune Disease

Compared with soluble factors directly secreted into the microenvironment, EVs confer bioactive stability to their cargo due to the presence of a protective membrane and improve bio-distribution of active molecules throughout the body. EVs have been indeed identified in biological fluids, including plasma, sperm, urine, milk and blood, demonstrating a systemic spreading of these nanocarriers in vivo. Growing evidence indicates that circulating EVs contribute to pathological processes, including cancer, inflammation and autoimmunity [53,54,55]. EV levels in body fluids have been also correlated with the disease course, as reported for cancer, including head, neck and ovarian cancer, where higher plasma levels of exosomes bearing tumor antigens have been found in patients with advanced disease stages compared with patients at the early stage of disease [56,57]. The spreading of self-antigen, as well as the elevated production of autoantibodies leading to the formation of immune complexes are key features of systemic autoimmune disease. In this context EVs bearing self-antigen and having the ability to protect and transfer their cargo at long distances might contribute to trigger and sustain pathological autoimmune responses.

### 4.1. Systemic Lupus Erythematosus

In systemic lupus erythematosus (SLE), a systemic autoimmune disease characterized by elevated levels of circulating anti-DNA antibodies, which ultimately lead to immune complex deposition and tissue destruction [58], a higher number of plasma EVs bearing immunoglobulins (Igs) G can be found compared with healthy controls, and interestingly, they correlate with anti-DNA antibody levels [59]. Although the cellular source of these EVs is unknown, apoptotic cell- and platelet-derived EVs displaying nuclear antigen, IgG and CD40 ligand have been recently suggested to be the major contributors of antinuclear autoimmunity in SLE [60,61]. Moreover in vitro generated EVs bearing antigenic determinants capable of binding anti-DNA antibodies and anti-nucleosome antibodies have been shown to interact with autoantibodies obtained both from lupus mice and SLE patients [62]. The presence of genomic DNA on EVs released by apoptotic cells has been recently demonstrated and suggested as a potential self-antigen in SLE. The deficiency of circulating deoxyribonuclease DNASE1L3, which is responsible for the clearance of cell-free DNA under homeostatic conditions, results indeed in enhanced levels of autoantibodies to DNA/chromatin and in the development of a lupus-like disease in mice [63]. Interestingly, Sisirak et al. [63] found that DNASE1L3 is the only circulating deoxyribonuclease capable of digesting chromatin on EVs. Loss of function of the secreted DNASE1L3 has been previously associated with SLE in both humans and mice [64,65]. 

Collectively, these data indicate a potential role of EVs as carriers of autoantigens and autoantibodies. Of note, apoptotic cell-derived EVs characterized by the exposure of galectin-3-binding protein (G3BP), which may interact with specific extracellular matrix proteins at the glomerular basement membrane, have been detected in the glomeruli of kidney biopsies of SLE patients where they colocalize with IgG [66], further supporting the pathogenic role of EVs in SLE autoimmunity. In agreement with the key role played by EVs in SLE and the potential of EVs to sustain the autoimmune response, we have documented an enhanced release of EVs from mast cells from mice lacking the *p66shc* gene, which are characterized by spontaneous mast cell and lymphocyte activation and the development of lupus-like autoimmunity [67,68]. Among Src homologous and collagen (Shc) protein A (SHCA), p66SHC is the longest isoform and negatively regulates TCR and B cell receptor (BCR) signaling pathways, thereby controlling lymphocyte activation and homeostasis and preventing autoimmunity [68]. Moreover, we have recently found that p66SHC controls mast cell degranulation and the release of EVs by inhibiting cytoskeletal dynamics through the stabilization of the SH2-containing inositol-5’-phosphatase 1 (SHIP-1) at the plasma membrane [69]. 

### 4.2. Multiple Sclerosis

Multiple sclerosis is an autoimmune disease of the central nervous system (CNS), where the disruption of the blood brain barrier (BBB) represents the incipit to disease development by favoring the migration of pathogenic lymphocytes into the CNS. This initial step is fundamental for the establishment of neuroinflammation, which is in turn responsible for neuron demyelination and the typical neurological manifestations. In this context, communication between endothelial cells, immune cells and CNS cells is fundamental first to allow lymphocyte infiltration into the CNS and then to regulate the function and stability of infiltrated autoreactive lymphocytes. 

While proinflammatory cytokines, such as tumor necrosis factor (TNF) α, interleukin (IL) -1β, interferon (IFN) γ and IL-17 released by circulating inflammatory cells, affect BBB integrity by directly disrupting tight junctions (IFNγ and IL-17), as well as by enhancing the activity of matrix metalloproteinase-9 (MMP-9) (IL-1β and TNFα) [70,71,72,73], EVs released from endothelial cells and platelets have been shown to increase endothelial permeability during MS [74] and to rapidly accumulate in the plasma of MS patients during disease relapses [75,76]. Among CNS cells, astrocytes and microglia, which release EVs containing metalloproteinases and IL-1β, have been also shown to contribute to BBB disruption [77,78,79]. Whether other proinflammatory cytokines are stored in EVs is presently not known. Interestingly, mice lacking acid sphingomyelinase (a-SMase), which are characterized by an impaired release of EVs from astrocytes and microglia, are protected from EAE, suggesting an important role for EVs in this disease [78,79,80]. It should however be pointed out that deficiency or inhibition of acid sphingomyelinase has been reported to impair the production of the pro-inflammatory cytokines IL-6 [81], as well as T cell transmigration across the brain endothelium [82]. Hence, the role of acid sphingomyelinase in EV release in MS remains to be conclusively established. Consistent with an important role played by EVs in MS, increased levels of EVs derived from oligodendroglial and microglial cells, correlating with disease course and severity, have been detected in the cerebrospinal fluid (CSF) both in EAE and in MS patients [83]. 

The release of EVs into the CSF by the choroid plexus epithelium has been recently documented and proposed as a novel mechanism of blood-brain communication [84,85]. Choroid plexus epithelium-derived EVs released into the CSF enter the brain parenchyma both under physiological conditions and upon systemic inflammation [84,85]. Interestingly, Balusu et al. [85] documented enhanced release of choroid plexus epithelium-derived EVs containing miRNA into the CSF upon systemic inflammation. These EVs were able to enter the brain parenchyma and promote inflammatory gene upregulation in astrocytes and microglia by transferring miRNAs [85]. In addition, primary human brain microvascular endothelial cell-derived EVs have been found to directly interact with effector CD4^+^ and CD8^+^ T cell through vascular cell adhesion molecule 1 (VCAM-1) and intercellular adhesion molecule 1 (ICAM-1) in vitro and to promote their proliferation by presenting antigen, as well as by expressing T-cell costimulatory molecules, including CD40 and inducible costimulator ligand (ICOSL) [86]. 

On the blood side of the BBB, the release of proinflammatory cytokines by effector T cells, mainly of the Th1 and Th17 subtypes, augments the expression of adhesion molecules on endothelial cells, thereby facilitating T cell adhesion and infiltration into the CNS. Activated T cells release moreover EVs containing CC chemokine ligand 5 (CCL5) and arachidonic acid, which may favor the recruitment of monocytes and sustain inflammation, a condition required for the disruption of the BBB integrity [76]. However, there is at present no direct evidence of a recruitment of monocytes to the BBB by T cell-derived exosomes.

Since microglia-derived EVs express major histocompatibility complex class II (MHCII) molecules on their surface, they are also likely to propagate neuroinflammation by restimulating CNS infiltrated encephalitogenic lymphocytes [87]. Moreover, enhanced release of microglia-derived EVs containing miRNA-155, which is upregulated in MS white matter microglia, has been suggested as an additional mechanism of microglia-mediated inflammation in this disease [88]. With the caveat that the transfer of EVs from microglia or other neural cells to T cells has not been demonstrated in vivo, the finding that miRNA-155 promotes the development of Th1 and Th17 cells and exacerbates EAE [45], taken together with the presence of this miRNA in microglia-derived EVs from MS patients, suggests that EVs carrying miRNA-155 and exposing MHCII on their surface are likely able to coordinately control the activation, effector function and stability of CNS-infiltrated Th17 and Th1 cells in MS.

The ability of EVs to cross brain barriers has been demonstrated in a mouse model using EVs carrying glyceraldehyde-3-phosphate dehydrogenase (GAPDH) small interfering RNA (siRNA), which following intravenous injection were able to specifically knock down the target gene in neurons, microglia and oligodendrocytes [89]. In addition, using transgenic mice expressing Cre-recombinase in the hematopoietic lineage, Ridder et al. [90] demonstrated that the transfer of functional RNA from blood cells to neurons occurs via EVs and is enhanced in response to peripheral inflammation, further supporting a crosstalk between hematopoietic cells and neural cells mediated by EVs. However, how EVs cross the brain barriers is still unknown and only recently, a model has been proposed based on evidence obtained from cancer cells and HIV-infected cells [91]. According to this two-stage model, during the first phase of this process, EVs internalized by the brain capillary endothelial cells release their cargo, including miRNA-150 and miRNA-181, which downregulates the expression of the tight junctions protein ZO-1 and of the 3-phosphoinositide-dependent protein kinase-1 (PDPK1) required for cofilin phosphorylation and actin polymerization, respectively, thereby affecting the integrity of tight junctions between endothelial cells and allowing a massive entry of EVs into the CNS in the second phase.

The EV-mediated crosstalk between hematopoietic cells and neural cells in MS strikingly demonstrates the key role played by EVs as nanocarriers in autoimmunity. In particular, the ability of EVs to cross the brain barriers and connect two microenvironments, the blood and the CNS, the latter being highly protected under normal conditions, together with their ability to spread neuronal antigens outside the CNS, makes EVs efficient carriers of information. These findings underline the importance of a better characterization of this form of cell-to-cell communication to better define the pathogenesis of MS and makes EVs attractive means both to monitor the activity of CNS cells by analyzing CNS-derived EVs in biological fluids and for the delivery of therapeutic proteins or nucleic acids to the CNS. The crossing of EVs from the blood to the brain and vice versa has been indeed documented. In this context, EVs obtained from blood samples of MS patients have been proposed as novel biomarkers for monitoring disease activity and response to therapy [83,88]. 

## 5. Conclusions

Collectively, these recent findings highlight EVs as an important source of self-antigens and immune complexes in autoimmune disease and provide evidence of the EV network as an efficient route for rapid dissemination of antigen, which might sustain and amplify the immune response to self-antigen, as well as potential innovative carriers for the horizontal transfer of nucleic acids (miRNA). EVs are also strongly emerging as key players in shaping the immune responses as the result of their ability to modulate the local microenvironment at the IS and to provide specific instructions to the APC in the form of cytokines and miRNAs. Depending on their cellular source, EVs carry at their surface a plethora of membrane-associated molecules that can modulate the response of immune cells, including TCR, MHC-associated peptide antigen, costimulatory and inhibitory receptors. As such, EVs have the potential to impinge on the differentiation, function and stability of Th subsets and to affect moreover the ability of Tregs to control effector T cells. 

The ability of autologous EVs to mimic an immune cell while lacking safety risks, as well as to preserve their cargo from degradation and dilution provide the rationale for the use of EVs as a safe and highly stable drug delivery system to treat autoimmune diseases. Although we are still far from a detailed characterization of EVs in the pathogenesis of autoimmune disease in vivo, the protective role of EV-mediated transfer in mouse models of autoimmune disease is promising. To use EVs as therapeutics, the major challenge we have to face is to understand how target cells take EVs up and whether this uptake is selective. Another major hurdle is the lack of a uniform nomenclature and a detailed characterization of EVs derived from different donor cells, which is the consequence of the exponential accumulation of data over the recent years from different research areas that until recently have not been interacting with each other, such that the large body of information is at present often heterogeneous, redundant and even misleading. Finally, a robust method to purify individual EV populations, which is a prerequisite for the uniform characterization of these vesicles, is still lacking. The current expansion of the research in this field, together with the development of novel isolation techniques and the efforts to unify both the already existing and the newly generated data will help to address the role of EVs in autoimmunity and to develop EV-based immunotherapy.
